# Big Data for Biomedical Education with a Focus on the COVID-19 Era: An Integrative Review of the Literature

**DOI:** 10.3390/ijerph18178989

**Published:** 2021-08-26

**Authors:** Rola Khamisy-Farah, Peter Gilbey, Leonardo B. Furstenau, Michele Kremer Sott, Raymond Farah, Maurizio Viviani, Maurizio Bisogni, Jude Dzevela Kong, Rosagemma Ciliberti, Nicola Luigi Bragazzi

**Affiliations:** 1 Clalit Health Services, Haifa & Western Galilee, Azrieli Faculty of Medicine, Bar-Ilan University, Safed 13100, Israel; rkhamisy@yahoo.com (R.K.-F.); peter.gilbey@biu.ac.il (P.G.); 2 Department of Industrial Engineering, Federal University of Rio Grande do Sul, Porto Alegre 90035-190, Brazil; leonardo.furstenau@ufrgs.br; 3 Business School, Unisinos University, Porto Alegre 91330-002, Brazil; sott.mk@gmail.com; 4 Department of Internal Medicine B, Ziv Medical Center, Azrieli Faculty of Medicine, Bar-Ilan University, Safed 13100, Israel; raymond.f@ziv.health.gov.il; 5 TransHumanGene, MedicaSwiss, 6330 Cham, Switzerland; info@transhumangene.com (M.V.); m_bisogni@hotmail.com (M.B.); 6 Laboratory for Industrial and Applied Mathematics (LIAM), Department of Mathematics and Statistics, York University, Toronto, ON M3J 1P3, Canada; jdkong@yorku.ca; 7 Section of History of Medicine and Bioethics, Department of Health Sciences (DISSAL), University of Genoa, 16132 Genoa, Italy; ciliberti@unige.it

**Keywords:** medical education, curriculum, big data, integrative review, COVID-19

## Abstract

Medical education refers to education and training delivered to medical students in order to become a practitioner. In recent decades, medicine has been radically transformed by scientific and computational/digital advances—including the introduction of new information and communication technologies, the discovery of DNA, and the birth of genomics and post-genomics super-specialties (transcriptomics, proteomics, interactomics, and metabolomics/metabonomics, among others)—which contribute to the generation of an unprecedented amount of data, so-called ‘big data’. While these are well-studied in fields such as medical research and methodology, translational medicine, and clinical practice, they remain overlooked and understudied in the field of medical education. For this purpose, we carried out an integrative review of the literature. Twenty-nine studies were retrieved and synthesized in the present review. Included studies were published between 2012 and 2021. Eleven studies were performed in North America: specifically, nine were conducted in the USA and two studies in Canada. Six studies were carried out in Europe: two in France, two in Germany, one in Italy, and one in several European countries. One additional study was conducted in China. Eight papers were commentaries/theoretical or perspective articles, while five were designed as a case study. Five investigations exploited large databases and datasets, while five additional studies were surveys. Two papers employed visual data analytical/data mining techniques. Finally, other two papers were technical papers, describing the development of software, computational tools and/or learning environments/platforms, while two additional studies were literature reviews (one of which being systematic and bibliometric).The following nine sub-topics could be identified: (I) knowledge and awareness of big data among medical students; (II) difficulties and challenges in integrating and implementing big data teaching into the medical syllabus; (III) exploiting big data to review, improve and enhance medical school curriculum; (IV) exploiting big data to monitor the effectiveness of web-based learning environments among medical students; (V) exploiting big data to capture the determinants and signatures of successful academic performance and counteract/prevent drop-out; (VI) exploiting big data to promote equity, inclusion, and diversity; (VII) exploiting big data to enhance integrity and ethics, avoiding plagiarism and duplication rate; (VIII) empowering medical students, improving and enhancing medical practice; and, (IX) exploiting big data in continuous medical education and learning. These sub-themes were subsequently grouped in the following four major themes/topics: namely, (I) big data and medical curricula; (II) big data and medical academic performance; (III) big data and societal/bioethical issues in biomedical education; and (IV) big data and medical career. Despite the increasing importance of big data in biomedicine, current medical curricula and syllabuses appear inadequate to prepare future medical professionals and practitioners that can leverage on big data in their daily clinical practice. Challenges in integrating, incorporating, and implementing big data teaching into medical school need to be overcome to facilitate the training of the next generation of medical professionals. Finally, in the present integrative review, state-of-art and future potential uses of big data in the field of biomedical discussion are envisaged, with a focus on the still ongoing “Coronavirus Disease 2019” (COVID-19) pandemic, which has been acting as a catalyst for innovation and digitalization.

## 1. Medical Education: An Overview of Its History and Evolution

Medical education refers to education and training delivered to medical students in order to become a practitioner [1].

The quickly evolving digital landscape, including the introduction of Artificial Intelligence in 1955, has profoundly modified and impacted the way medical education is provided [2,3]. Chan and Zary [4] have carried out an integrative review of the peer-reviewed literature and have included and synthesized 37 articles, finding that Artificial Intelligence has been utilized for three major purposes in the field of medical education: namely, (I) supporting and enhancing students’ learning experience; (II) assessing and monitoring educational/learning outcomes; and (III) reviewing and improving the medical school curriculum (especially the undergraduate one). Artificial intelligence can enable direct and personalized/individualized interactions between the instructor and the students, facilitating exchanges and feedback, better guiding, informing, and customizing the learning pathway. Not only can the educational transactions happen in real-time, but they are also cost-effective, given the decreased costs generated by the use of Artificial Intelligence [5].

However, as for other information and communication technologies innovations, the full development and implementation of Artificial Intelligence in the medical educational system has been proven to be technically challenging, requiring the instructors to devise novel methodological frameworks and approaches to ensure data privacy and integrity, avoiding cheating and big data manipulation [6].

Distance education—known also as distance learning, distributed learning, e-learning, or online learning—consists in delivering courses to students not always physically present at school [7]. Courses can be indeed conducted by means of classical face-to-face lectures in the classroom or utilizing a hybrid/blended approach, exploiting the new information and communication technologies and immersive environments (known also as virtual realities or virtual worlds). Computer-enhanced lessons, such as massive open online courses (known as MOOCs), differ from traditional residential courses, in that they are offered to larger-scale communities of students and usually require/enable a much more interactive participation. According to Schneider and Germann (1999) [8], distance learning has evolved during the decades, passing from mail correspondence and print materials (the so-called phase of ‘correspondence study’, or the first generation of distance learning) to voice, mail, fax, radio, television, video, and audio recordings with interactive instructions (the phase of ‘multimedia distance teaching’ or ‘broadcast/teleconferencing’, or the second generation of distance learning). Further evolutions include emails, digital ‘serious games’, webinars, webcasts/podcasts (the phase of ‘interactive, web-based instruction’, or the third generation of distance learning) [9].

The successful evolution of digital learning environments (i.e., eClass, CourierSera or Moodle) driven also by open-source and reusable software, has profoundly transformed the experience and the processes associated with the education and training of future professionals, including medical doctors. Students often make use of such environments and platforms for study and exam preparation, acquiring practical information—what is called experiential or immersive learning [10].

Big data are characterized by five Vs: namely, (I) velocity (referring to the speed and the pace at which they are generated); (II) volume (related to the unprecedented amount of data available); (III) variety (because they are inherently heterogeneous); (IV) veracity (referring to the data quality, accuracy, and reliability); and (V) value (when big data are properly processed and analyzed and become actionable, smart data). Based on the sources and channels that produce and release big data, they can be classified/categorized into: computational, digital, and molecular (generated by wet-lab, next-generation sequencing, and high-throughput technologies) [11,12]. Big data are leading innovation and designing a new era, the so-called disruptive or innovative era [11,12].

In recent decades, medicine has been radically transformed by scientific and computational/digital advances, including the introduction of new information and communication technologies, the discovery of DNA, the birth of genomics and post-genomics super-specialties (transcriptomics, proteomics, interactomics, and metabolomics/metabonomics, among others), which contribute to the generation of an unprecedented amount of data—the so-called big data [13]. While these are well-studied in fields such as medical research and methodology, translational medicine, and clinical practice, they remain overlooked and understudied in the field of medical education [14,15]. Therefore, in the present paper, we will review the current and potential future applications of big data with a special focus on the field of medical education and the still ongoing “Coronavirus Disease 2019” (COVID-19) outbreak [16]. Besides straining healthcare facilities worldwide, this pandemic is posing challenging safety issues, both for instructors and professors and students who should be able to safely deliver and receive lectures, respectively, while, at the same time, integrity and continuity of the medical education process should be guaranteed. Access to education is, indeed, a fundamental right, which has been seriously threatened and limited/restricted during the pandemic: biomedical education is of crucial importance to train and form future specialists.

On the other hand, schools, and universities closures, one of the “Non-Pharmaceutical Interventions” (NPIs) that have been implemented and enforced by health authorities, have been effective in curbing the number of infections and flattening the epidemic curve [17]. However, besides disrupting educational systems, COVID-19 has also propelled transformation and digitization, acting as catalyst for innovations and accelerating the adoption of technologies and new strategies/approaches.

Big data represent an innovative tool that can be exploited to enhance and improve biomedical education. However, to the best of our knowledge, there is a lack of information concerning the potential roles of big data in the biomedical educational field, especially during the COVID-19 pandemic.

As such, this review was designed to fill in this gap of knowledge. More specifically, we decided to focus on emergent technologies’ disruptive effects on biomedical education contextualized in the COVID-19 era. This review can have practical implications for all the relevant actors and stakeholders involved in the process of delivering medical education, including decision- and policy-makers.

## 2. Material and Methods

A comprehensive integrative review of the literature was conducted, mining one of the major scholarly electronic biomedical databases (namely, PubMed/MEDLINE). The search string utilized comprised of several components, one related to medical school (medical students, medical residents, specialists), one to education (curriculum, syllabus), and one to big data. More in detail, the search string was: “big data” AND (“medical education” OR “medical school” OR “school of medicine” OR “medical syllabus” OR “medical faculty” OR “faculty of medicine” OR “medical degree” OR “medical degree program curriculum”) AND (“resident” OR ”residents” OR “residency” OR “specialist” OR “specialists” OR “specialization” OR “student” OR “students” OR “undergraduate” OR “graduate” OR “postgraduate” OR “trainee” OR “trainees”). The search string was devised together with an expert health librarian.

Inclusion criteria were: (I) articles concerning the use of big data in the field of medical education; (II) original investigations of any approach (experimental, quasi-experimental, or non-experimental/theoretical) as well as reviews (of any type, narrative, systematic, integrative, etc.), editorials, letters to the editor, commentaries, technical notes, expert opinions, clinical case reports or case series; and, finally, (III) studies written in English language. Exclusion criteria were as follows: (I) studies not related to the use of big data in the field of medical education; and, (II) studies not written in English language. No time filters were applied: databases were mined from inception.

References of potentially eligible studies were reviewed to increase the chance of getting potentially relevant studies and curbing the odds of missing some important works in the field.

An integrative review is one of the 14 types of reviews delineated by the “Search, Appraisal, Synthesis and Analysis” (SALSA) committee [18]. More specifically, it is a general review of the existing scholarly literature, conceived as a “systematic” process that leads to the synthesis and integration of several (either quantitative or qualitative) studies, in an inclusive fashion. The execution of an integrative review consists of five stages/steps: namely, (I) problem/research question identification and formulation; (II) comprehensive literature search; (III) data retrieval, extraction, and collection; (IV) data processing and analysis; and (V) discussion, future prospects, and conclusions [19].

A multi-disciplinary team was set up, with three experts in medical education (R.K.-F., P.G., and R.C.); an expert in research methodology (N.L.B.), two experts in literature reviews (L.B.F., and M.K.S.), an expert in internal medicine (R.F.), and three experts in big data and artificial intelligence (M.V., M.B., and J.D.K.).

Two authors independently (L.B.F. and M.K.S.) searched the scholarly literature and extracted relevant items to be included in the present integrative review. In case of disagreement, a third author (N.L.B.) was consulted, who acted as final referee. Themes were identified and categorized by three authors independently (R.K.-F., P.G., and R.C.), using an ad hoc devised Excel spreadsheet. In case of disagreement, consensus was reached through discussion or consulting a fourth author (N.L.B.). The latter process was carried out employing the Sandelowski’s qualitative content analysis theoretical framework [20], which implied: (I) familiarizing with the literature included and getting a sense of the whole; (II) extracting all the relevant facts; (III) identifying and stressing storylines, topics, and content; and, finally, (IV) utilizing data reduction frameworks.

## 3. Results

**Figure 1:**
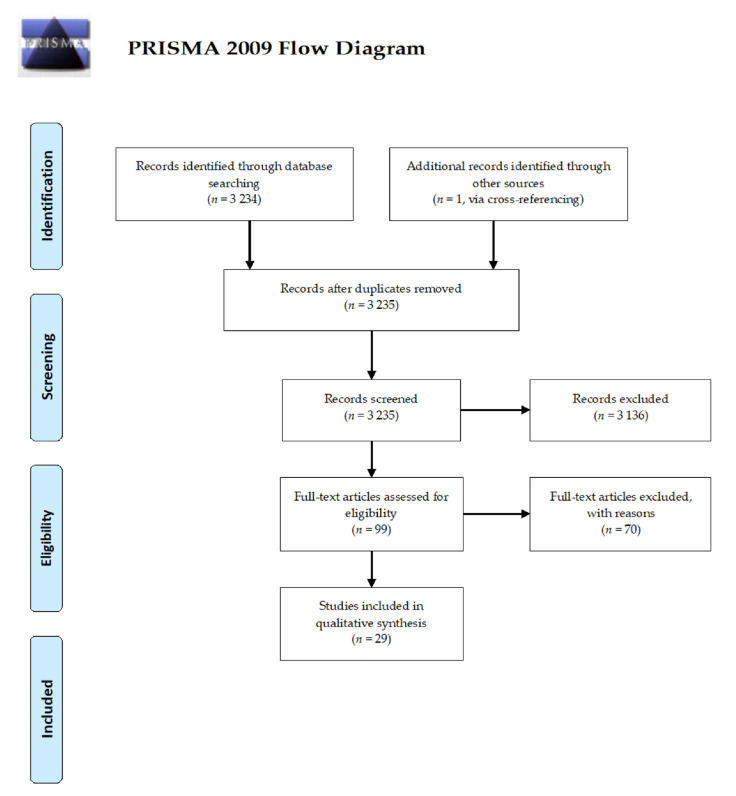
Flowchart showing the retrieval and inclusion processes strategy adopted in the present integrative review.

The initial search yielded a pool of 3,234 items. One additional item was retrieved via cross-referencing. A subset of 3,136 articles were excluded being irrelevant to the topic under study, when reading the title and/or the abstract. A set of 99 studies were read in full text, and 70 were excluded with reasons, after reading their full text. Finally, based on the previously mentioned inclusion/exclusion criteria, 29 studies were included in the present integrative review, as pictorially shown in Figure 1.

Included studies were published between 2012 and 2021. Eleven studies were performed in North America: specifically, nine were conducted in the USA and two studies in Canada. Six studies were carried out in Europe: two in France, two in Germany, one in Italy, and one in several European countries. One additional study was conducted in China. Eight papers were commentaries/theoretical or perspective articles, while five were designed as a case study. Five investigations exploited large databases and datasets, while five additional studies were surveys. Two papers employed visual data analytical/data mining techniques. Finally, two other papers were technical papers, describing the development of software, computational tools and/or learning environments/platforms, while two additional studies were literature reviews (one of which being systematic and bibliometric).

The following major nine topics could be identified: (I) knowledge and awareness of big data among medical students; (II) difficulties and challenges in integrating and implementing big data teaching into the medical syllabus; (III) exploiting big data to review, improve, and enhance medical school curriculum; (IV) exploiting big data to monitor the effectiveness of web-based learning environments among medical students; (V) exploiting big data to capture the determinants and signatures of successful academic performance and counteract/prevent drop-out; (VI) exploiting big data to promote equity, inclusion, and diversity; (VII) exploiting big data to enhance integrity and ethics, avoiding plagiarism and duplication rate; (VIII) empowering medical students, improving and enhancing medical practice; and, (IX) exploiting big data in continuous medical education and learning. These sub-themes were subsequently grouped in the following four major themes/topics: namely, (I) big data and medical curricula; (II) big data and medical academic performance; (III) big data and societal/bioethical issues in biomedical education; and, (IV) big data and medical career (Table 1).

### 3.1. Knowledge and Awareness of Big Data among Medical Students

As incorporating Artificial Intelligence into the medical profession, to enable future medical practitioners to gain a better understanding of Artificial Intelligence-based algorithms, integrating the teaching of big data into the medical syllabus is of paramount importance as well, given their increasing role in the field of biomedicine. Recently, the “Italian Young Medical Doctors Association” [21] has carried out a web-based survey to quantitatively explore the levels of digital health- and big data-related awareness and knowledge among young medical doctors. For the purpose, authors assessed the following areas/topics: computational big data (predictive and nowcasting/forecasting models), digital big data (including Artificial Intelligence, Internet of Things (IoT), Internet of Medical Things (IoMT), social media and social networks, blockchain, clinical data storing and processing systems, and tele-medicine), molecular big data (OMICS approaches and high-throughput technologies, such as next-generation sequencing and micro-arrays). Authors were able to sample 362 subjects, only 6–22% of which reporting experience with at least one type of big data during their clinical or study/research activities. Based on the participants’ replies, authors identified three profiles of medical student/resident: namely, high-, low-, and no-tech. These findings warrant the incorporation of lectures dedicated to digital health and big data to enrich and enhance medical education.

Similarly, in North America, more specifically in Canada, Chow-White et al. [22] explored the knowledge of genomics in a sample of medical oncologists from the federal province of British Columbia, finding a low to moderate level of literacy. Interestingly, 42% of the recruited sample stated that the genomic training offered by the medical programs was not enough. These results suggest the need to improve the knowledge level about genomics and other molecular big data among medical students and residents.

### 3.2. Integrating and Implementing Big Data Teaching into the Medical Syllabus: Difficulties and Challenges

Paralleling the progress and evolution of information and communication technologies, medicine has evolved becoming more and more complex (systems biology and systems/network medicine, precision, and personalized medicine). Despite such a complexity, medical students are not well-equipped in terms of tools and conceptual frameworks. As pointed out by Hoy [23], courses should be dedicated to disciplines like data-science, biomathematics and biostatistics, information science and bioinformatics [24], reflecting the urgent and contemporary need for a multi-disciplinary teaching, at the intersection of various specialties and knowledge branches. Students should be provided with courses focusing more on quantitative skills, incorporating machine learning and artificial intelligence, mathematical modeling, computation and simulation, and big data [25].

This is true both at the undergraduate and postgraduate level, with several medical specializations (like radiology, oncology, physical medicine, rehabilitation, rheumatology, cardiology, or urology, among others) expanding current knowledge levels, skills, and competencies [26,27]. Attempts to redesign specialist syllabus are very recent, but it can be anticipated that they will have a great impact on future medical practice. The “Multi-Institutional Academic Trainee Interpretation Log Database” (MATILDA) is an important example of a big data-based initiative among radiology residents [28].

To facilitate the process of integration and incorporation, scientific biomedical knowledge has to be coherently organized into a unified framework, able to explain a disease at different levels (namely, etiopathogenesis, structural changes, physiopathology, and clinical manifestations). Sánchez Fernández de la Vega [29] has proposed a new conceptual framework, termed as “Human Structural Biopathology”.

### 3.3. Exploiting Big Data to Review, Improve, and Enhance Medical School Curriculum

Big data can be utilized also for reviewing medical school curriculum, which is the major tool that instructors and course directors have in order to devise and deliver teaching, assess and grade students’ activities, and track and monitor its effectiveness in terms of learning outcomes. Since there is an increasing need for a highly qualified and trained healthcare personnel, which is even more urgent in light of the still ongoing “Coronavirus Disease 2019” (COVID-19) pandemic, when healthcare facilities have faced shortage of workforce, medical classes are usually large and comprise of a high number of students. As such, medical curricula and associated teaching/learning pathways and experiences are characterized by the generation of a big amount of data, which is difficult to handle and analyze with classical data manipulation techniques. Vaitsis et al. [30,31] have utilized big data and Information Analytics and Visualization-based tools to identify eleven aspects underlying the development of medical school curricula, which have been grouped in three major categories: namely, (I) teaching methodologies and related learning outcomes; (II) students’ activities and examinations, assessment, and grading; and (III) current gaps in medical education. The approach elaborated by Vaitsis et al. [30,31] paves the way to a new super-specialty within medical education, termed as “medical education informatics”, which can inform in an evidence-based and data-driven fashion the development and implementation of healthcare syllabuses.

Similarly, Fritze et al. [32] have exploited big data to improve and enhance medical curricular transparency in terms of coverage, sequence and consistence of competencies and learning outcomes, monitoring and assessment of learning objectives. For this purpose, authors devised a web-based, interactive, user-friendly platform (MERlin database) enabling instructors to map, diagnose and develop curricular activities and students to visualize and better understand them. Four German medical faculties pilot-tested the database, which is currently used by 14 out of 38 German faculties. Authors concluded that the tool implemented is easy to use and understand, facilitates the development of goal- and competencies-oriented curricular syllabuses, based on national standards, enables to share experiences and benchmarks.

### 3.4. Exploiting Big Data to Monitor the Effectiveness of Web-Based Learning Environments among Medical Students

Big data and educational data mining techniques can be used to track and monitor the effectiveness of web-based learning environments and adaptive learning platforms, as done by Menon et al. [33] in a sample of US medical students utilizing an environment named Osmosis, in the study period August 2014–July 2015.

Similarly, Luo et al. [34] utilized big data to explore medical students’ ability and self-confidence with self-learning and learning environments in a sample of 116 students from two grades. Authors were able to confirm the effectiveness of such platforms.

### 3.5. Exploiting Big Data to Capture the Determinants and Signatures of Successful Academic Performance and Counteract/Prevent Drop-Out

Baron et al. [35] exploited big data to capture signatures of successful academic performance among medical undergraduate students. “Paths to success” were modeled using a set of 53 parameters extracted from admissions application records of 1088 students at the New York University School of Medicine in the period 2006–2014. Four, different clusters of medical students could be identified: the “risers”, the “improvers”, the “solids”, and the “statics”, based on the combination of admission and subsequent scores. More specifically, the “risers” and the “statics” reported the highest and lowest chances of academic success. Authors concluded that the medical student population is not homogenous, but inherently heterogeneous and that a big data-based approach can effectively identify the determinants of academic performance related outcomes and potentially counteract/prevent drop-out, which imposes a significant societal and economic-financial burden.

Similar findings were replicated by Bientzle et al. [36]. Utilizing big data generated by 23,633 users using Amboss, an online website-based platform, in the period April 2014–April 2017, authors assessed if particular formats of learning activities and materials (multiple choice questions versus learning cards or individual notes) were related to learning outcomes. It was fond that online platforms facilitating experiential, immersive learning are particularly effective in that they are able to engage the medical student and improve their knowledge level.

### 3.6. Exploiting Big Data to Promote Equity, Inclusion, and Diversity

While training medical doctors and forming future professionals, it is also crucial to educate them about the values and asset of equity, inclusion, and diversity [37]. In recent decades, racism and discrimination against minorities have been rampant, also in multicultural societies. It is an onus of the educational bodies and systems to make efforts to be more inclusive and to support minority trainees and early-career physicians. Big data analytics can help policy- and decision-makers as well as educational professionals track and monitor the effectiveness of their affirmative programs and interventions, to build a more just and fair society [37].

### 3.7. Exploiting Big Data to Enhance Integrity and Ethics, Avoiding Plagiarism and Duplication Rate

Big data coupled with Artificial Intelligence can be effectively used to screen scholarly articles under peer-review as well as students’ exam-related material, submitted for assessment and grading [38]. Integrity and ethics issues have, indeed, become more relevant during the COVID-19 era with the transition from in-presence to remote, online examination [39].

### 3.8. Empowering Medical Students, Improving and Enhancing Medical Practice

Similarly to what has happened and is still happening with Artificial Intelligence, medical education can take advantage of big data to enhance and enrich the learning experience of students. There exist several online/web-based, data-driven applications developed specifically for medical students and/or residents who can practice diagnosing diseases or recognizing co-morbidities and their impact on prognosis. For instance, TrainCoMorb [40], relying on dynamic Bayesian algorithms and on a big claims database (comprising of 11 million data), enables medical trainees to predict the attributable risk for negative health-related outcomes, simulating, for example, the likelihood of death, developing a hospital septicemia or other healthcare-associated infections. Like TrainCoMorb, other big data-based medical applications and tools are expected to help medical undergraduate students as well as specialists in training have a better understanding of the various clinical scenarios, exploring the effect of each socio-demographic/clinical variable on health outcomes.

These applications are particularly useful to increase awareness of rare diseases and clinical phenomena, such as the Raynaud phenomenon, pointing out to ‘red flags’, uniquely combining experiential learning, role-play simulation, workshops, and big data. Some trials conducted have shown the effectiveness of such interventions, which have significantly improved medical students’ learning outcomes [41]. However, further, high-quality research in this field is needed.

Other applications and ‘serious games’ can help future practitioners better understand major public and global health challenges, such as antimicrobial resistance. AntibioGame®, for instance, has been shown to be useful to teach medical students a correct antibiotic prescription [42], suggesting that gamification and big data can be integrated in a multi-faceted medical training syllabus.

Kang et al. [43] assessed the outcomes of the “Value of Imaging Through Comparative Effectiveness Research Program” (VOICE), which provided residents in diagnostic imaging, radiology, and cardiology with education in research methodology and big data analytics, through hands-on, practical lectures.

However, the precise relationship between medical education and patient care in terms of health outcomes remains to be elucidated yet. Professor Dr. Marc Triola [44], Associate Dean for Educational Informatics, Associate Professor of Medicine at the New York University Langone, and Founding Director of the “Institute for Innovations in Medical Education” (IIME), is doing research aimed at filling the gap in knowledge concerning medical syllabus, educational reforms, and professional practice. For this purpose, he is utilizing data from around 8,500 graduate students of the New York University School of Medicine, and approximately 12,000 graduates of the New York University Langone’s residency training programs.

Very few studies have attempted to explore the link between pedagogical approaches to medical education and training (for example, among residents) and health outcomes. Cobb et al. [45] have conducted a study to assess whether resident participation in five high-risk elective general surgery procedures may result in serious concerns in terms of post-operative outcomes (30-day mortality rate, post-operative infections, and operative time), mining the “American College of Surgeons National Surgical Quality Improvement Program” (ACS-NSQIP) database in the period 2005–2012. Authors utilized advanced statistical techniques, such as machine learning (more specifically, decision trees) and found that surgical resident participation was safe in terms of mortality and/or complications.

Rajkomar et al. [46] exploited big data to quantitatively evaluate the effectiveness of clinical immersion in core rotations among internal medicine residents at an academic medical center in the period July 2012–June 2014, by extensively mining electronic health records generated by 10,022 hospitalizations of patients suffering from 1436 different diagnoses.

Recently, Chahine et al. [47] have described a grassroots initiative, inclusive of university institutions from one Canadian federal province and national licensing organizations which are working very closely to acquire, pre-process, link, and analyze big data to investigate the association between medical education, professional practice, and patient healthcare outcomes, in such a way to inform policy- and decision-makers, regulators, medical educators and managers, as well as researchers.

Finally, Feldman and Chawla [48] suggested to use large databases, like the Centers for Medicare & Medicaid Services (CMS) Medicare release to understand the effect of previous experiences at the medical school on clinical daily practice.

### 3.9. Exploiting Big Data in Continuous Medical Education and Learning

Medical doctors never stop learning and acquiring new information. Medicine is a discipline continuously under flux: for this reason, continuous medical education helps professionals and practitioners maintain an adequate level of competences, skills, and literacy, besides enabling them to learn about new and developing/emerging areas in their field/specialization. In that regard, Au-Yong-Oliveira et al. [49] have conducted a systematic, bibliometric review of the literature and have found four major themes: namely, (I) “data transformation”-related events underlying the process of medical learning, through medical system; (II) “health intelligence” that informs and guides the medical doctors in learning about health innovations via making predictions; (III) “data leveraging” that enables to learn from patient’s information; and, (IV) “clinical decision-making” in diagnosing diseases, delivering treatment, and managing the patients. In this way, big data can boost and enhance the medical learning processes, conjugating knowledge, research, and innovation.

## 4. Discussion

Big data are having an increasing role in the field of medical education, despite the fact that they remain understudied. Big data applications have been more studied in fields such as medical research and methodology, translational medicine, and clinical practice.

Big data can further enhance medical education, by digitalizing and personalizing/customizing it, adapting the teaching and delivery of lectures to the individual needs of each medical student, rather than being a ‘one size fits it all’ solution to educational requests [50].

Medical education is not without costs. In the USA, a recent National Academy of Medicine report called for reforms to how graduate medical education is provided and financed. Harnessing Big data can be helpful in reviewing the cost-effectiveness of some pedagogical approaches and enhancing the teaching/learning experiences [51].

The still undergoing COVID-19 has shown the importance of a qualified, well-trained healthcare personnel. Many hospitals and health facilities have been overwhelmed by COVID-19 cases, with the infectious agent imposing a considerable stress and strain on the system, worldwide. This has impacted as well learning and teaching, disrupting the educational system due to the implementation of NPIs such as social distancing [52,53,54,55,56].

Learning is a dynamic, interactive process. More specifically, medicine/medical learning represents a complex ‘learning health system’, in which the medical student/resident/doctor continuously learns from interacting with the instructor/patient, respectively. Big data can facilitate and assist these processes, guiding and informing them. However, despite the enormous production of big data in biomedicine, medical education remains centered on information acquisition and application processes [57]. A transition towards “knowledge discovery”, “knowledge management”, and “knowledge application”, in such a way that big data become actionable and operational data, is absolutely necessary, even if it is highly challenging [57]. A shift towards a “competency-based medical educational system” is also of paramount importance [58,59].

To develop a real learning health system, the different ‘cultures’ and ‘languages’ underlying it have to communicate and interact with each other in an inter-, multi-disciplinary perspective [58,60]. Boundaries have to be crossed and the gaps have to be filled [58].

Big data can facilitate and support this ‘cross-cultural’ talking and provide a lot of data at the individual, institutional, sub-national (regional or provincial), national and global level. Big data can act as a catalyst for discoveries and innovations, acting on each stage and level of medical education, both at the “upstream” and “downstream” level [61,62,63,64,65].

## 5. Conclusions and Future Perspectives

Big data in Medical Education remain overlooked and understudied, despite their promising role. The unique, unprecedented convergence of disciplines, such as machine learning and artificial intelligence, computational simulations, and mathematical modeling, data science and big data offers new opportunities to accelerate and expedite the pace of scientific discoveries and scholarly advances in the field of biomedicine (Table 2). However, current biomedical curricula and syllabuses appear inadequate to prepare future medical professionals and practitioners that can leverage on big data in their daily, clinical practice. COVID-19 is acting as a catalyst for innovation, accelerating the implementation of several changes related to the delivery of medical lectures and the need for more integrated, open curricula.

## Figures and Tables

**Table 1:** Main characteristics of the studies included in the present integrative review of the literature.

Reference	Year	Study Design	Country	Main Findings	Main Topic	Sub-Topic
Casà et al. [21]	2021	Web-delivered, questionnaire-based study, with 362 subjects (young medical doctors)	Italy	Only 6–22% out of 362 participants had knowledge of big data. Replies to the survey enabled to profile three types of medical doctor: namely, high-tech, low-tech, and no-tech	Big data and medical curricula	Knowledge and awareness of big data among medical students
Chow-White et al. [22]	2017	Survey-based study, with 31 participants (medical oncologists, 52.5% response rate)	British Columbia, Canada	Knowledge was moderate. Participants perceived and anticipated that big data are likely to have a great impact on medical education and practice in the future		
Hoy [23]	2021	Commentary	Not applicable	Advanced statistical courses (data analysis/data science) should be offered, as well as homegrown bootcamps and workshops, taking advantage of open-source and freely available online materials (such as YouTube tutorials)		Integrating and implementing big data teaching into the medical syllabus: difficulties and challenges
Capdarest-Arest and Navarro [24]	2021	Case study	Blaisdell Medical Library, University of California, Davis/Sacramento, California, USA	There is a need for data-related training in biomedical education. Health science libraries can help develop and implement integrated syllabuses		
Robeva et al. [25]	2021	Commentary/theoretical article/perspective article	Not applicable	Open curricula and syllabuses integrating modeling methods, experimental, and computational approaches through engaging, problem-oriented, and hands-on projects, fully exploiting the pedagogy of “experiential teaching” and “experiential learning”. Utilizing big data in medical education implies a major paradigm shift		
Zanca et al. [26]	2021	Case study	Several European countries (Belgium, the Netherlands, Italy, Spain, France, Ireland, Greece, Switzerland, Germany, Finland, Norway)	Authors devised an ad hoc curriculum for medical physicists (medical imaging and radiation therapy) incorporating big data and artificial intelligence components and utilizing a knowledge, skills, and competences (KSC) approach		
Hameed et al. [27]	2021	Literature review	Not applicable	Big data can enrich and enhance curricula of residents in urology and related sub-specialties (urologic oncology, renal transplant, urolithiasis, reproductive urology, pediatric urology, and endourology)		
Chen et al. [28]	2016	Case study	USA	MATILDA is an important example of a big data-based initiative among radiology residents from three American diagnostic radiology residency programs (one in the South, one in the Midwest, and a third in the Northeast of the USA)		
Sánchez Fernández de la Vega [29]	2020	Conceptual paper	Not applicable	A proposal of an updated system of the Organization of Scientific Biomedical Knowledge		
Vaitsis et al. [30,31]	2014	Visual analytics-based study	Not applicable	Authors identified three approaches of educational data visualization and representation and five competencies at undergraduate medical program level addressed in courses		Exploiting big data to review, improve and enhance medical school curriculum
Fritze et al. [32]	2019	Design-based, multicenter study	Germany	Authors devised and implemented a web-based interactive platform for medical curriculum mapping, diagnostics, and development (MERlin database)		
Menon et al. [33]	2017	Educational data mining-based study, with a sample of 6787 medical students	USA	Authors used a big data-based approach to investigate the determinants of the use of an adaptive learning platform called Osmosis	Big data and medical academic performance	Exploiting big data to monitor the effectiveness of web-based learning environments among medical students
Luo et al. [34]	2015	Technical paper (software/platform development and effectiveness case-control study, recruiting a sample of 116 medical students)	The Second Military Medical University Shanghai, Shanghai, China	Authors found that big data can provide strategic information and facilitate decision-making in improving medical students’ self-learning skills		
Baron et al. [35]	2020	Database-based study, with a sample of 1088 medical students	New York University Grossman School of Medicine, New York, NY, USA	Authors utilized “academic big data” (standardized tests and undergraduate grade point average or uGPA) to identify various paths to academic success, via an optimized logistic regression model. Authors identified four different paths/trajectories to success		Exploiting big data to capture the determinants and signatures of successful academic performance and counteract/prevent drop-out
Bientzle et al. [36]	2019	Database-based study, with a sample of 23,633 students	Germany	Authors exploited big data to link between use of an online platform (Amboss) providing learning materials for preparation for medical exams and performance outcomes		
Argueza et al. [37]	2021	Commentary/theoretical article/perspective article	Not applicable	Authors provided recommendations, including use of big data, to make contemporary society more inclusive and less racist	Big data and societal/bioethical issues in biomedical education	Exploiting big data to promote equity, inclusion, and diversity
Levin et al. [38]	2020	Commentary	Not applicable	Authors showed how to exploit big data to counteract ethical breaches, avoid plagiarism and preserve research integrity in the field of medical education and research		Exploiting big data to enhance integrity and ethics, avoiding plagiarism and duplication rate
Madhavanprabhakaran et al. [39]	2021	Commentary	Not applicable	Authors showed the difficulties and barriers to the implementation of remote teaching in the field of nursing education and how COVID-19 acted as a catalyst for innovation		
Zikos et al. [40]	2020	Technical paper (software development)	Not applicable	Authors described the development of TrainCoMorb, an online, data-driven (claims database-based) application specifically devised for medical students and residents. The application can help students recognize comorbidities and understand their prognostic outcome by means of a dynamic Bayesian algorithm	Big data and medical career	Empowering medical students, improving and enhancing medical practice
Sange et al. [41]	2020	Case-study	France	Authors showed how big data can be utilized to raise awareness of rare symptoms/diseases, via two simulated consultations Participants felt very satisfied with the training and more comfortable about diagnosing and managing rare symptoms/diseases. Big data, role-playing, simulations, “immersive learning” and involvement of patient educators, together with other innovative, emerging educational tools, can play a significant role in medical education		
Tsopra et al. [42]	2020	Questionnaire-based study, administered to 57 medical students	France	AntibioGame^®^ is a case-based serious game, which enables medical students to simulate various real-life primary care scenarios. It was deemed good and quite satisfactory in terms of usability and playability		
Kang et al. [43]	2018	Case-study	USA	Authors described the development of the Value of Imaging Through Comparative Effectiveness (VOICE) Research Program, which provides medical residents from radiology and cardiology with practical, hands-on training in five areas, including big data principles, and applications of big data analytics, and exploits blended teaching		
Triola and Pusic [44]	2012	Literature review and case-study	USA	Authors described the development of the Education Data Warehouse, an initiative aimed at utilizing big data to link between medical educational curricula and patient healthcare outcomes		
Cobb et al. [45]	2018	Database-based study, with a sample of 25,363 patients	USA	Authors exploited big data to monitor the safety of surgical operations performed by medical residents		
Rajkomar et al. [46]	2017	Retrospective, database-based study	University of California, San Francisco, USA	Authors analyzed 53,066 clinical notes from 10,022 hospitalizations with 1436 different diagnoses spanning 217 diagnostic categories, written by postgraduate year 1, 2, and 3 internal medicine residents. Educational gaps could be identified		
Chahine et al. [47]	2018	Literature review and case-study	Ontario, Canada	Authors presented a grassroots initiative, showing that big data can be used to dissect the relationship between various pedagogical/educational approaches to medical training and patient healthcare outcomes		
Feldman and Chawla [48]	2015	Database-based analysis (geographic distribution analysis, and school similarity analysis)	USA	Authors utilized a large database to link between medical doctor’s training and performance/clinical outcomes. Big data can be exploited to shed light on knowledge transfer and educational experiences		
Au-Yong-Oliveira et al. [49]	2021	Systematic literature review and bibliometric review of two major scholarly databases (PubMed/MEDLINE and Google Scholar), carried out by means of VOSviewer software	Not applicable	215 studies were included, with a total of 757 (co-)authors. Fifteen authors resulted particularly inter-connected. Similarly, 379 out of 569 keywords were highly inter-connected. Based on the review, authors formulated a 4-dimension model: (I) data transformation (health information services; new health applications; medical images; artificial intelligence systems related to health procedures); (II) health intelligence (epidemic outbreak forecasting/nowcasting; drug discovery; big data analytics applied to diseases prediction; genome data); (III) data leveraging (patient information; health records; operational data; public health data); and, (IV) decision-making (disease diagnosis; real-time monitoring and tracking of patients; improving and enhancing of medical treatments; offering and delivering of patient-centered care provisions)		Exploiting big data in continuous medical education and learning

**Table 2:** Uses and applications of big data in the field of biomedical education.

Potential Use/Application (Example)
Empowering medical students and enhancing classical education
Counteracting drop-out
Digitalizing education
Personalizing education
Promoting equity, inclusion, diversity
Enhancing integrity and ethics, avoiding plagiarism and duplication rate
Enhancing medical practice and continuous medical education and learning

## Data Availability

All data are available within the manuscript.
